# Effect of Exercise on Kidney Function, Oxidative Stress, and Inflammation in Type 2 Diabetic KK-*A^y^* Mice

**DOI:** 10.1155/2012/702948

**Published:** 2012-07-26

**Authors:** Yuji Ishikawa, Tomohito Gohda, Mitsuo Tanimoto, Keisuke Omote, Masako Furukawa, Saori Yamaguchi, Maki Murakoshi, Shinji Hagiwara, Satoshi Horikoshi, Kazuhiko Funabiki, Yasuhiko Tomino

**Affiliations:** Division of Nephrology, Department of Internal Medicine, Juntendo University Faculty of Medicine, 2-1-1 Hongo, Bunkyo-ku, Tokyo 113-8421, Japan

## Abstract

Exercise is recommended for the management of type 2 diabetes, but its effects on diabetic nephropathy (DN) are still unknown. We hypothesized that appropriate exercise improves early DN via attenuation of inflammation and oxidative damage. Type 2 diabetic KK-*A* ^*y*^ mice, a spontaneous DN model, underwent two different kinds of exercise (i.e., moderate and low intensity). Sedentary mice or those undergoing an exercise regimen causing no significant body weight loss were used. We examined the urinary excretion of albumin, number of podocytes and macrophages, renal expressions of HIF-1**α** and MCP-1, and biomarkers of oxidative stress such as urinary 8-OHdG and serum SOD. Exercise reduced urinary levels of albumin and also maintained the number of podocytes in the exercised KK-*A* ^*y*^ mice independently of improvements of overweight and hyperglycemia, although moderate-intensity exercise increased expression of HIF-1**α**. Sedentary KK-*A* ^*y*^ mice showed increased expression of MCP-1 and infiltration of macrophage, increased urinary 8-OhdG, and decreased serum SOD levels compared with exercised KK-*A* ^*y*^ mice. On the whole, low-intensity exercise attenuates progression of early DN without affecting marked renal ischemia. Reduction rates of urinary albumin and maintained podocyte numbers, with parallel improvements in oxidative damage and inflammation, are related to beneficial effects of exercise in diabetic kidney disease.

## 1. Introduction

Recent studies suggest that a chronic inflammatory process and oxidative stress promote the progression of diabetic nephropathy (DN) [1–4]. We have also showed the presence of macrophage infiltration and increased MCP-1 expressions and levels in glomeruli and urine of KK-*A* ^*y*^ mice, a frequently used animal model of type 2 diabetes (T2D) [5, 6]. Furthermore, urinary 8-OHdG, a marker of oxidative DNA damage, was also increased in this mouse model [7]. 

Lifestyle modification, especially appropriate exercise, is recommended for the management of T2D through improvements of metabolic risk factors such as blood pressure, blood glucose, plasma lipids, and oxidative stress markers. On the other hand, this also consumes considerable amounts of oxygen, leading to production of high levels of reactive oxygen species (ROS). There is also evidence that ROS and high glucose exposure contribute to podocyte apoptosis in experimental DN [8]. It is considered that exercise-induced proteinuria is usually not permanent but evanescent [9, 10]. Moreover it is little known that moderate exercise has adverse effect on the renal function [11–14]. Several studies reported that exercise showed renoprotective effects in both types of diabetic subjects as well as in animal models of DN, although detailed mechanism of action by which exercise has a favorable influence on renal function is not yet fully understood [15–18].

In 1969, the KK-*A* ^*y*^ mouse was established by Nishimura [19]. This mouse was produced by the transfer of the yellow obese gene (*A* ^*y*^ allele) into the KK mouse. Because the diabetic feature in the KK-*A* ^*y*^ mouse is more severe than that in the KK mouse, this mouse is widely used as an experimental model for T2D. KK-*A* ^*y*^ mice spontaneously exhibit T2D associated with hyperglycemia, glucose intolerance, hyperinsulinemia, obesity, and microalbuminuria. Renal lesions in KK-*A* ^*y*^ mice closely resemble those in human DN with glomeruli exhibiting diffuse mesangial hyperplasia with mesangial cell proliferation, segmental sclerosis, and overexpression of TGF *β*-1 at 20 weeks of age. We have reported that the KK-*A* ^*y*^ mouse is a suitable model for the study of DN in humans [6, 7, 20–22].

 In the present study, we hypothesized that appropriate exercise may improve early DN via attenuation of the expression of inflammation and oxidative stress in the kidneys of KK-*A* ^*y*^ mice.

## 2. Materials and Methods

### 2.1. Experimental Animals and Protocols

Eight-week-old male diabetic KK-*A* ^*y*^/Ta Jcl mice were purchased from CLEA Japan (Tokyo, Japan). We also purchased the same age male KK/Ta Jcl mice as a control for the KK-*A* ^*y*^ mouse. The mice were individually housed in plastic cages with free access to food (rodent pellet diet NMF; 348 kcal/100 g, containing 5.5% crude fat) and water throughout the experimental periods. All mice were maintained in the same room under conventional conditions with a regular 12-hour light/dark cycle and temperature controlled at 24 ± 1°C. All experiments were performed according to the guidelines of the Animal Care Committee of Juntendo University.

After acclimating to new surroundings and running on a treadmill (Osaka Micro System, Osaka, Japan) for 4 weeks, KK-*A* ^*y*^ mice were divided into three groups: (1) sedentary group (no exercise), (2) low-intensity exercise group, and (3) moderate-intensity exercise group (*n* = 10–13 per group). Mice were exercised at 10 m/min for an hour 5 days a week in the moderate-intensity group or 5 m/min for 30 minutes 3 days a week in the low-intensity group for 8 weeks. Nonexercised KK-*A* ^*y*^ and KK mice were placed on nonrotating wheels for the same duration. The experimental protocol was terminated when the mice reached 20 weeks of age.

### 2.2. Biochemical Measurements

The urinary albumin-creatinine ratio (ACR), body weight (BW), and hemoglobin A1c (HbA1c) were measured at 12 and 20 weeks of age. The levels of urinary 8-hydroxydeoxyguanosine (8-OHdG), N-acetyl-*β*-D-glucosaminidase (NAG), serum creatinine, superoxide dismutase (SOD), and creatinine clearance (Ccr) were measured at 20 weeks of age. Urinary samples were collected for 24 hours using a metabolic cage (mouse metabolic cage, CLEA Japan). Urinary albumin and creatinine samples were measured by immunoassay (DCA 2000 System; Bayer Diagnostics, Elkhart, IN). Glucose levels in blood obtained from the retroorbital sinus were measured using Glucocard (Kyoto Daiichi Kagaku, Kyoto, Japan). HbA1c was also measured by immunoassay (DCA 2000 system, Bayer Diagnostics). Serum creatinine was measured by an autoanalyzer (Fuji Dry-chem 5500; Fujifilm, Tokyo, Japan). Urinary 8-OHdG level was measured by an enzyme-linked immunosorbent assay (Fushimi Pharmaceutical, Kagawa, Japan) [23]. Serum SOD activity (SOD Assay Kit-WST, Dojindo Molecular Technologies, Tokyo, Japan) was measured by enzyme immunoassay. Ccr was estimated as the ratio of daily urinary creatinine excretion to plasma creatinine concentration and then expressed as milliliters per minute per square meter of surface area.

### 2.3. Immunohistochemical Staining for Hypoxia Inducible Factor (HIF)-1*α*, Monocyte Chemotactic Protein (MCP)-1, CD68, and CD204

The mice were sacrificed at 20 weeks of age. The kidneys were retrogradely perfused with saline via the abdominal aorta for 5 minutes at a pressure of about 150 mmHg without prior flushing of the vasculature. Immunostaining of the frozen sections or paraffin-embedded sections was performed. Renal tissues were snap frozen in an optimal cutting temperature compound and cut into 3-*μ*m-thick sections. Formalin-fixed and paraffin-embedded tissues were cut at 2 *μ*m. Immunohistochemical studies were performed using commercially available antibodies as follows: mouse monoclonal anti-HIF-1*α* antibody (ab-1; Abcam, MA, USA), goat polyclonal anti-MCP-1 antibody (sc-1784; Santa Cruz, CA, USA), rat monoclonal anti-CD68 (the marker for pan macrophage) (MCA1957; AbD Serotec, Oxford, UK), and rat monoclonal anti-CD204 (the marker for M2 macrophage) (MCA1322; AbD Serotec, Oxford, UK). HIF-1*α* stained sections were then blocked with mouse Ig blocking reagent (MKB-2213; Vector Laboratories, CA, USA). The other stained sections were blocked by blocking solution (2% fetal bovine serum and 10% normal goat serum in PBS). The sections were incubated with secondary antibody: anti-goat IgG, anti-mouse IgG, and anti-rat IgG, respectively (Nichirei, Tokyo, Japan). Secondary antibody was visualized by light microscopy with diaminobenzidine.

The HIF-1*α* staining of at least 20 randomly selected fields (×200) from each mouse was quantified using the KS-400 version 4.0 image analysis system (KS-400; Carl Zeiss Vision, Munich, Germany). The numbers of M1, M2, and MCP-1-positive cells were counted in at least 20 randomly selected fields (×200) and/or at least 20 glomeruli. Analyses were performed by two investigators in a blinded fashion [6, 24].

### 2.4. Quantitative Analysis of MCP-1 mRNA by Real-Time PCR

Real-time PCR was also used to evaluate MCP-1 mRNA expression in whole kidneys of mice at 20 weeks of age. Total RNA was extracted from whole kidneys using the RNeasy Mini Kit (Qiagen K.K., Tokyo, Japan). Complementary DNA was synthesized using random hexamers (Quantum RNA kit; Ambion, Austin, Tex., USA). The complementary DNA was further amplified by a real-time polymerase chain reaction (PCR) system (ABI Prism 7500 Real-Time PCR System; PerkinElmer, Foster City, Calif., USA). Initial template concentration was derived from the cycle number at which the fluorescent signal crosses a threshold in the exponential phase of the PCR reaction. Relative gene expression was determined based on the threshold cycles (Ct values). The PCR parameters were 95°C for 10 min, 50 cycles at 95°C for 15 s, and 60°C for 60 s. Primers and fluorogenic probes of MCP-1 were obtained from TaqMan Gene Expression Assays, Applied Biosystems (Foster City, Calif., USA). The assay identity number of MCP-1 was Mm-00441242-m1 and GAPDH was Mn-99999915-g1.

### 2.5. Immunofluorescent Staining for Podocytes (WT-1)

Immunofluorescent staining for podocytes in renal tissues was performed using the polyclonal rabbit anti-mouse WT-1 antibody (Santa Cruz, SC-192). Goat anti-rabbit Alexa Fluor 488 was used to visualize WT-1 positive cells. The sections were mounted with a fluorescent mounting media (Dako Cytomation) before visualization with a Fluoview 1000 confocal microscope (Olympus, Tokyo, Japan) and FV10-ASW software (version 1.3c; Olympus).

The staining of at least 20 glomeruli from each mouse was quantified using the KS-400 image analysis system. The number of podocytes per glomerular area was determined using the method of Weibel [25]. The ratio of podocyte number to glomerular area was also calculated. These examinations were performed by two investigators without knowledge of the origin of the slides, and then mean values were calculated.

### 2.6. Statistical Analysis

Data were expressed as mean ± SD. Statistical differences between means were determined using Dunnett's test. A value of *P* < 0.05 was considered to be statistically significant.

## 3. Results

### 3.1. Biochemical Parameters

Before the start of the experiment, we measured the baseline fasting blood glucose, body weight, and urinary albumin of each animal at 12 weeks of age, and confirmed that there were no significant differences in those values among sedentary and exercised KK-*A* ^*y*^ mice. However, these parameters in sedentary KK-*A* ^*y*^ mice were significantly higher compared with those in sedentary KK mice (Table 1).

**Figure 1:**
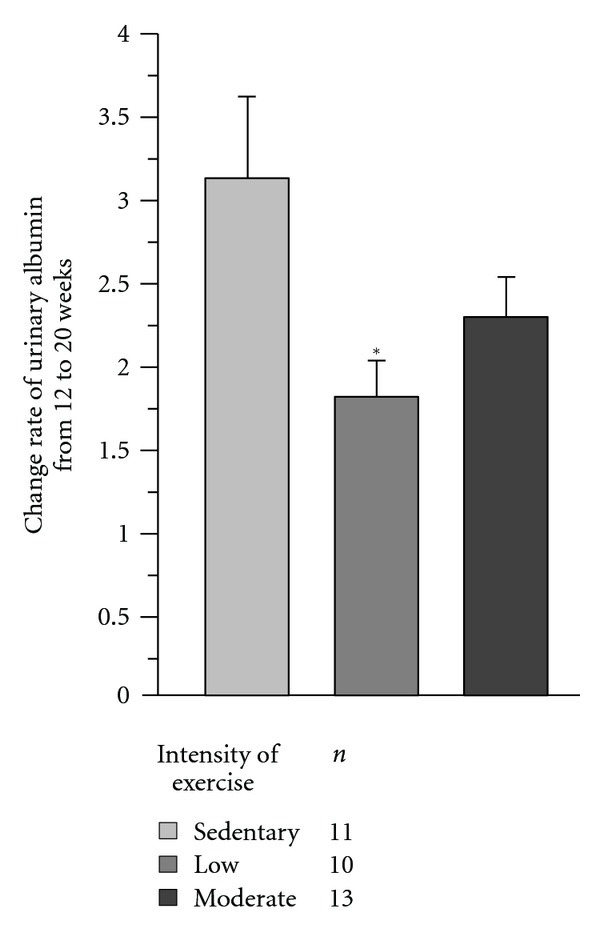
Change rate of urinary albumin from 12 to 20 weeks in KK-*A* ^*y*^ mice. Change rate of urinary albumin at 20 weeks of age divided by that at 12 weeks of age. Low-intensity exercised KK-*A* ^*y*^ mice had greater change rate of urinary albumin compared with sedentary mice. *P < 0.05.

 Biochemical parameters after the 8-week experimental protocol are also shown in Table 1. Body weight and HbA1c levels in sedentary KK-*A* ^*y*^ mice remained at a high level compared with those in sedentary KK mice. However, these levels did not differ among sedentary and exercised KK-*A* ^*y*^ mice. The levels of Ccr in KK-*A* ^*y*^ mice were slightly increased compared with those in KK mice. However, there were no statistically significant changes in the levels of Ccr between sedentary KK-*A* ^*y*^ mice and sedentary KK mice. Moreover, no significant changes were observed in the levels of Ccr between sedentary and exercised KK-*A* ^*y*^ mice (Table 1). Urinary albumin levels in the exercised KK-*A* ^*y*^ mice tended to be lower than those in sedentary KK-*A* ^*y*^ mice, although the difference was not statistically significant. However, the rate of urinary albumin change from 12 to 20 weeks (Figure 1) and urinary NAG levels in KK-*A* ^*y*^ mice with low-intensity exercise reached statistically significant levels. Exercised KK-*A* ^*y*^ mice had significantly lower levels of urinary 8-OHdG and higher serum SOD compared with sedentary KK-*A* ^*y*^ mice. These levels approached those of KK mice, although these did not differ between low- and moderate-intensity exercised KK-*A* ^*y*^ mice.

### 3.2. Immunohistochemistry of HIF-1*α*, MCP-1, CD68, and CD204 in the Kidneys

**Figure 2:**
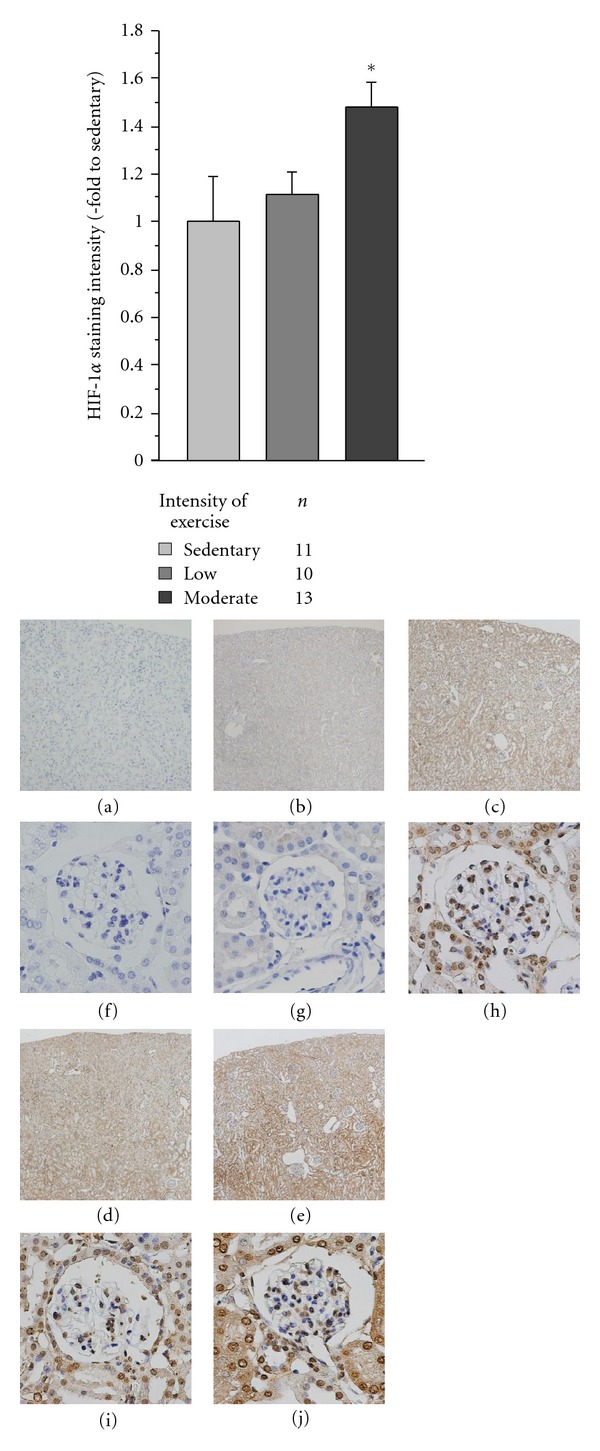
HIF-1*α* staining intensity and staining in KK-*A* ^*y*^ mice with and without exercise at 20 weeks of age. HIF-1*α* staining intensity in moderate-intensity exercised KK-*A* ^*y*^ mice was significantly enhanced compared with that in the sedentary KK-*A* ^*y*^ mice, but these levels did not differ between sedentary and low-intensity exercised KK-*A* ^*y*^ mice. *P < 0.001. Representative HIF-1*α* staining in the renal cortex of sedentary KK (b, g), sedentary KK-*A* ^*y*^ (c, h), low-intensity exercised KK-*A* ^*y*^ (d, i), and moderate-intensity exercised KK-*A* ^*y*^ mice. (e, j) Staining (a, f) depicts the absence of primary antibody. Images (a–e) were taken at 40-fold magnification and images (f–j) were taken at 400-fold magnification. *P < 0.001.

HIF-1*α* proteins were mainly localized in the tubules and interstitium adjacent to the medulla. Contrary to expectations, the expression of HIF-1*α* in the moderate-intensity exercised KK-*A* ^*y*^ mice was significantly higher compared with that in sedentary KK-*A* ^*y*^ mice, although it did not differ between sedentary and low-intensity exercised KK-*A* ^*y*^ mice (Figure 2). Also, HIF-1*α* expression was not observed in sedentary KK mice.

**Figure 3:**
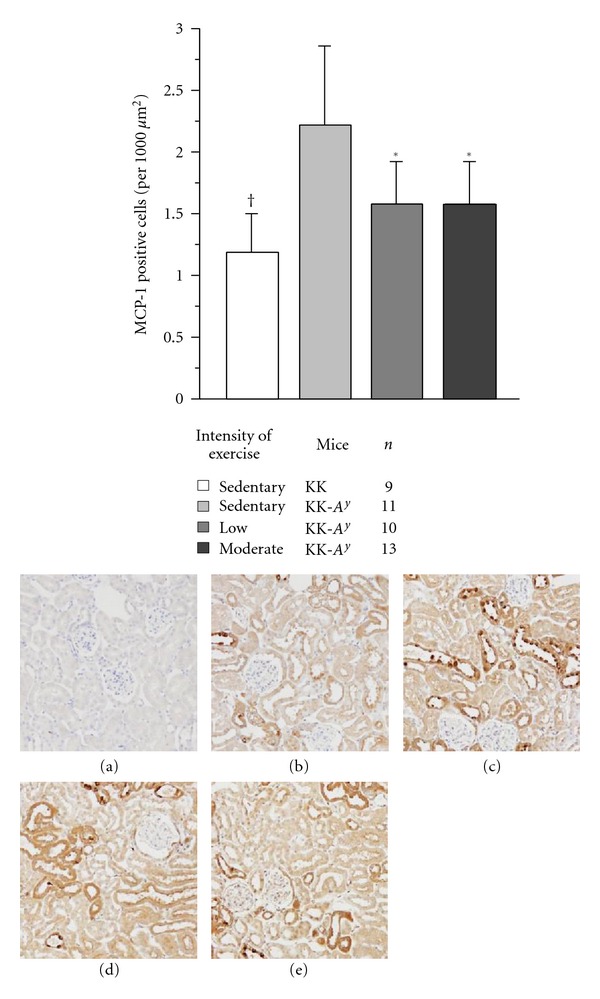
Number of MCP-1 positive cells and staining in each mouse at 20 weeks of age. The number of MCP-1 positive cells in sedentary KK-*A* ^*y*^ mice was significantly greater compared with that in the exercised KK-*A* ^*y*^ mice and also sedentary KK mice. Representative MCP-1 staining in the renal cortex of sedentary KK (b), sedentary KK-*A* ^*y*^ (c), low-intensity exercised KK-*A* ^*y*^ (d), and moderate-intensity exercised KK-*A* ^*y*^ mice. (e) Staining (a) depicts the absence of primary antibody. Images were taken at 100-fold magnification. *P < 0.05^†^ *P* < 0.001.

MCP-1 positive cells were localized in the proximal tubules. The numbers of MCP-1 positive cells per 1000 *μ*m^2^ in the exercised KK-*A* ^*y*^ mice were significantly lower compared with those in the sedentary KK-*A* ^*y*^ mice, and came close to those of the sedentary KK mice (Figure 3).

**Figure 4:**
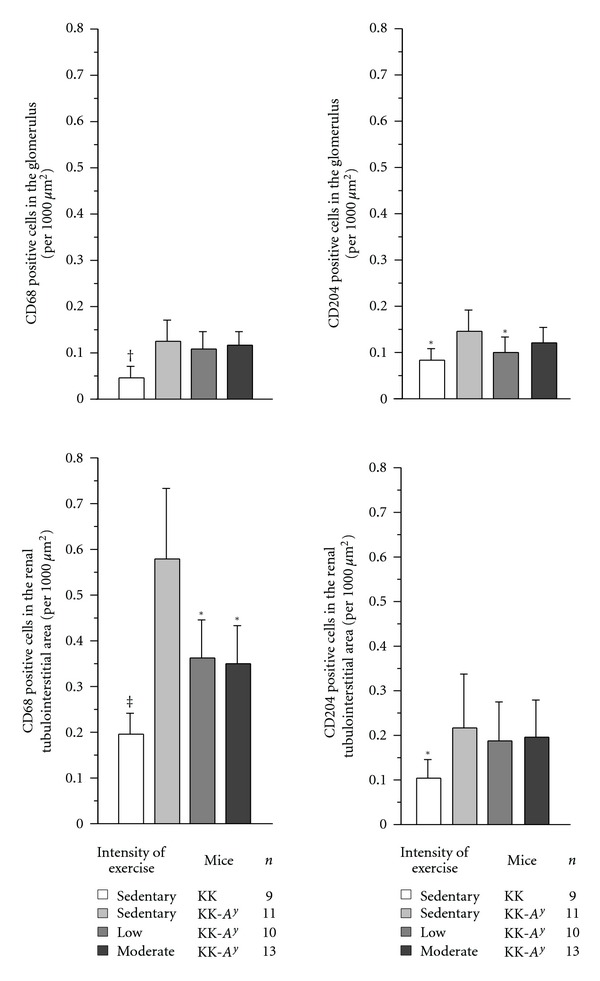
Number of CD68 and CD204 positive cells and staining in each mouse at 20 weeks of age. Bar charts show CD68 and CD204 positive cells in the glomeruli and tubulointerstitial area of sedentary KK (b), sedentary KK-*A* ^*y*^ (c), low-intensity exercised KK-*A* ^*y*^ (d), and moderate-intensity exercised KK-*A* ^*y*^ mice. (e) The number of CD68 positive cells in glomeruli of sedentary KK-*A* ^*y*^ mice were significantly greater compared with that of sedentary KK mice, but it did not differ between sedentary and exercised KK-*A* ^*y*^ mice. The number of CD68 positive cells in glomeruli of sedentary KK-*A* ^*y*^ mice was significantly greater compared with that of sedentary KK mice and also low-intensity exercised KK-*A* ^*y*^ mice, but not moderate-intensity exercised KK-*A* ^*y*^ mice. The number of CD68 positive cells in the tubulointerstitial area of sedentary KK-*A* ^*y*^ mice was significantly greater compared with that of sedentary KK mice and also exercised KK-*A* ^*y*^ mice, but it did not differ between low- and moderate-intensity exercised KK-*A* ^*y*^ mice. The number of CD204 positive cells in tubulointerstitial area of sedentary KK-*A* ^*y*^ mice was significantly greater compared with that of sedentary KK mice, but it did not differ between sedentary and exercised KK-*A* ^*y*^ mice. *P < 0.05 ^†^ *P* < 0.01 ^‡^ *P* < 0.001.

The number of M2 macrophages in the renal tubulointerstitial area was not changed between sedentary and exercised KK-*A* ^*y*^ mice, but that of estimated M1 macrophages, calculated by subtracting the number of CD204 from CD68 positive cells in intensely exercised KK-*A* ^*y*^ mice, showed a significant decrease compared to the sedentary KK-*A* ^*y*^ mice. Although the number of CD68 and CD204 positive cells was similar in the glomeruli, the number of CD68 and CD204 positive cells in the glomeruli was much lower than that in the renal tubulointerstitial areas. This result suggests that most of macrophages are considered to be M2 macrophages in the glomeruli, and that the number of M2 macrophages showed a reduction in the low-intensity exercised KK-*A* ^*y*^ mice as compared to the sedentary KK-*A* ^*y*^ mice (Figure 4).

### 3.3. Renal Expression of MCP-1 mRNA in the Kidney

**Figure 5:**
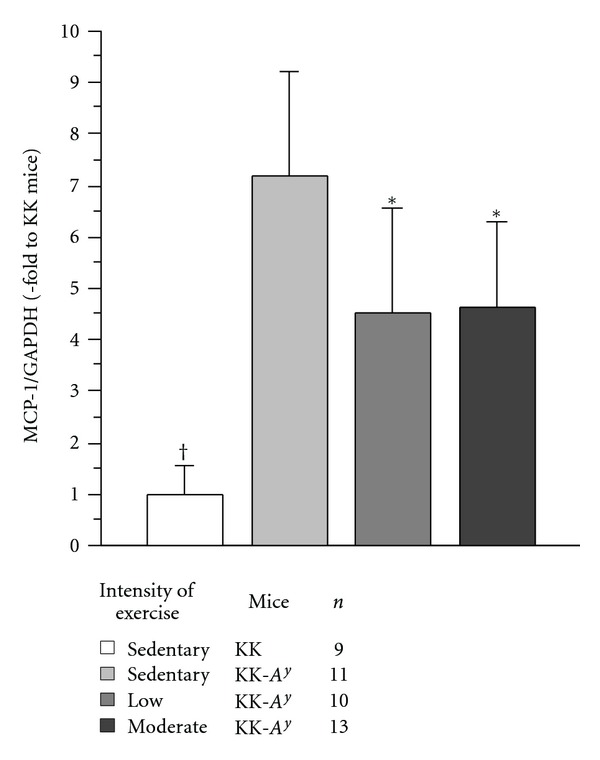
Renal expression of MCP-1 mRNA in the kidney at 20 weeks of age. The level of MCP-1 mRNA l in the sedentary KK-*A* ^*y*^ mice was significantly higher than that in exercised KK-*A* ^*y*^ mice and also sedentary KK mice. *P < 0.05 ^†^ *P* < 0.01.

As shown in Figure 5, the level of MCP-1 mRNA l in the sedentary KK-*A* ^*y*^ mice was significantly higher than exercised KK-*A* ^*y*^ mice and also sedentary KK mice.

### 3.4. Number of WT-1 Positive Cells

**Figure 6:**
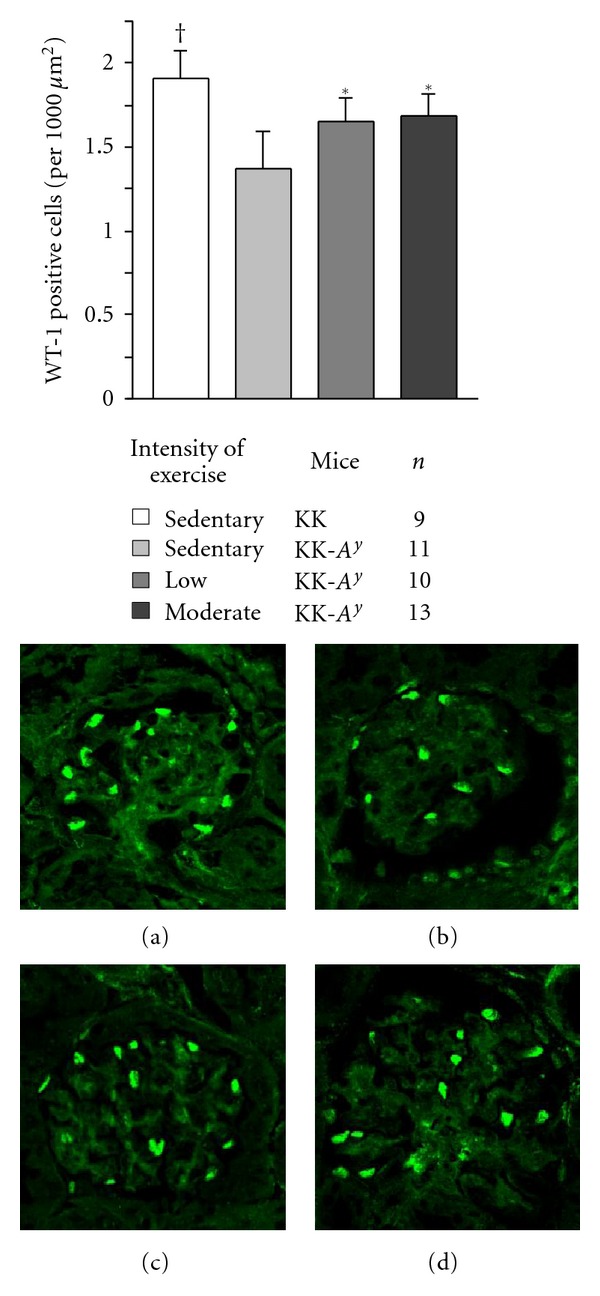
Number of WT-1 positive cells and staining in each mouse at 20 weeks of age. WT-1 positive cells in exercised KK-*A* ^*y*^ mice were significantly maintained compared with those in the sedentary KK-*A* ^*y*^ mice and approached to those in KK mice. *P < 0.01 ^†^ *P* < 0.001. Representative WT-1 staining in the renal cortex of sedentary KK (a), sedentary KK-*A* ^*y*^ (b), low-intensity exercised KK-*A* ^*y*^ (c), and moderate-intensity exercised KK-*A* ^*y*^ mice. (d) Images were taken at 400-fold magnification.

As shown in Figure 6, the number of glomerular WT-1 positive cells, that is podocytes, per 1000 *μ*m^2^ of glomerular areas in the sedentary KK-*A* ^*y*^ mice was significantly lower than that in sedentary KK mice. On the other hand, the number of podocytes in low- and moderate-intensity exercised KK-*A* ^*y*^ mice was significantly greater than that in the sedentary KK-*A* ^*y*^ mice.

## 4. Discussion

In the present study, if the body weight of exercised mice did not increase compared with that of sedentary mice when mice were allowed to eat ad libitum, we defined such exercise as moderate intensity. We also demonstrated that exercise training improved urinary NAG levels as well as the change rate of urinary albumin from 12 to 20 weeks, independent of body weight and glycemic status in the kidneys of KK-*A* ^*y*^ mice, although moderate-intensity exercise increased expression of HIF-1*α* in the kidneys. In this study, no significant changes were observed in the levels of Ccr between sedentary KK-*A* ^*y*^ and exercised KK-*A* ^*y*^ mice. Therefore, it is indicated that the decrease of urinary albumin was not due to the reduction of renal blood flow/glomerular filtration rate, but more likely to the effect of exercise. Our running program (10 m/min for one hour, 5 days a week for a total of 8 weeks) was very similar but slightly less intense compared with the program of Huang et al. (15 m/min for 45 min, 5 days a week, for a total of 8 weeks) [26]. Interestingly, they also demonstrated that exercise training does not affect body weight in KK-*A* ^*y*^ mice although the starting time of exercise differed from that in our protocol (12–20 weeks versus 8–16 weeks).

There are several mechanisms for the renoprotective effects of exercise in DN. In general, exercise training ameliorates renal function by improving metabolic factors such as plasma lipids, blood glucose, blood pressure, and body weight. It is also known to improve renal histology without altering metabolic factors in line with our present study. Boor et al. [27] demonstrated that exercise training reduced advanced glycation end products (AGEs) in both serum and kidney tissues of obese Zucker rats, an animal model of T2D and associated nephropathy, without altering inflammatory biomarkers or metabolic factors. In contrast, our study clearly showed that the exercised mice showed attenuated renal expression of MCP-1 and infiltration of macrophage in the kidneys. In general, macrophage activation is defined into two polarizations, M1 and M2, in adipose tissues [28], although the implication of these macrophages in DN is not yet fully understood. The M1 macrophage produces proinflammatory cytokines, such as TNF*α* and IL-6, while on the other hand, the M2 macrophage produces an anti-inflammatory cytokine [29]. The number of macrophages in glomeruli and the tubulointerstitial area increased with the progression of DN. The pattern of M1 macrophage in tubulointerstitial areas was similar to that of MCP-1 positive cells, suggesting that exercise might attenuate MCP-1 expression by preventing M1 macrophage infiltration, mainly in the tubulointerstitial areas. In glomeruli, contrary to our expectations, most of macrophages seemed to be M2 macrophages, and these macrophages increased with the progression to DN, although the number of pan macrophages in the glomeruli was very small compared with that in tubulointerstitial areas. It is still unknown whether the function of M2 macrophages in the kidney is different from that in adipose tissues or if M2 macrophages just increase to compensate for the increase of M1 macrophage in the kidney. Further study will be required to explain this.

It is considered that appropriate exercise increases antioxidant enzymes, although excessive exercise causes inflammation, increases oxidative stress associated with ROS, and decreases renal blood flow and glomerular filtration rate. Moien-Afshari et al. [30] demonstrated that expression of SOD isoform depends on exercise intensity in the aorta of diabetic db/db mice. In the present study, both intensities of exercise increased serum SOD levels, even though we did not confirm each isoform level. Moreover, both exercises decreased urinary 8-OHdG level, an oxidative stress marker. However, contrary to our expectation, low-intensity exercise was more effective than moderate-intensity exercise in terms of renal function. We require further investigation to determine appropriate exercise intensity. Although flawed, this study indicated that exercise training might attenuate podocytopenia and albuminuria, partly through anti-inflammatory or/and anti-oxidant effects because it did not affect metabolic factors. 

The main limitations of our study are as follows. We did not measure blood pressure, and the beneficial effects of exercise might be partly derived from antihypertensive effects. Furthermore, we could not estimate appropriate exercise intensity in human from these results. 

In conclusion, low-intensity exercise attenuates progression of early diabetic nephropathy without affecting marked renal ischemia. However, attention should be paid to renal ischemia even though albuminuria improved. Reductions in rate of urinary albumin change, urinary NAG, and maintained podocyte numbers, with parallel improvements in oxidative damage and chronic inflammation, might be related to beneficial effects of exercise in diabetic kidney disease.

## Figures and Tables

**Table 1:** Biochemical parameters in each mouse at 12 and 20 weeks of age.

	KK	KK-*A* ^*y*^	KK-*A* ^*y*^	KK-*A* ^*y*^
Number	9	11	10	13
Intensity of exercise	Sedentary	Sedentary	Low	Moderate
12 weeks of age				
Body weight (g)	31.9 ± 0.6^‡^	39.9 ± 2.4	40.9 ± 2.2	40.3 ± 2.1
HbA1c (%)	4.0 ± 0.1^‡^	7.0 ± 0.9	7.4 ± 1.1	6.7 ± 1.4
Urinary albumin (mg/g·Cr)	86 ± 120^‡^	398 ± 203	434 ± 177	402 ± 160
20 weeks of age				
Body weight (g)	36.7 ± 1.2^‡^	44.4 ± 3.6	46.3 ± 2.9	44.5 ± 3.3
FBG (mg/dL)	97 ± 15	87 ± 29	72 ± 19	91 ± 25
HbA1c (%)	4.4 ± 0.4^‡^	8.8 ± 1.1	9.5 ± 1.3	8.4 ± 1.3
Serum creatinine (mg/dL)	0.14 ± 0.06	0.16 ± 0.05	0.15 ± 0.05	0.16 ± 0.05
Urinary albumin (mg/g·Cr)	98 ± 43^‡^	1104 ± 569	726 ± 388	833 ± 370
Urinary NAG (U/mg/L·Cre)	9.7 ± 2.0^‡^	22.8 ± 6.0	16.7 ± 4.4*	17.3 ± 3.5
Urinary 8-OHdG (*μ*g/g·Cre)	47 ± 11^†^	306 ± 159	56 ± 42^†^	97 ± 105^†^
Serum SOD (U/dL)	243 ± 101	226 ± 37	316 ± 40^†^	326 ± 59^‡^
Ccr (mL/min)	64.7 ± 12.3	88.8 ± 33.9	82.9 ± 29.7	84.2 ± 36.4

Data expressed as means ±  SD. *P < 0.05 versus KK-*A* ^*y*^ sedentary, ^†^ *P* < 0.01 versus KK-*A* ^*y*^ sedentary,^  ‡^ *P* < 0.001 versus KK-*A* ^*y*^ sedentary.

HbA1c: hemoglobin A1c, FBG: fasting blood glucose, NAG: N-acetyl-*β*-D-glucosaminidase, 8-OHdG: 8-hydroxydeoxyguanosine, SOD: super oxide dismutase, and Ccr: creatinine clearance.

## A list of Abbreviations

AGEs: Advanced glycation end products
ACR: Albumin-creatinine ratio
BW: Body weight
Ccr: Creatinine clearance
DN: Diabetic nephropathy
HbA1c: Hemoglobin A1c
HIF: Hypoxia inducible factor
MCP: Monocyte chemotactic protein
NAG: N-acetyl-*β*-glucosaminidase
ROS: Reactive oxygen species
SOD: Superoxide dismutase
T2D: Type 2 diabetes
8-OHdG: 8-hydroxydeoxyguanosine.
